# Investigation of anterior cingulate cortex gamma-aminobutyric acid and glutamate-glutamine levels in obsessive-compulsive disorder using magnetic resonance spectroscopy

**DOI:** 10.1186/s12888-019-2160-1

**Published:** 2019-05-30

**Authors:** Yan Li, Chen Cheng Zhang, Kathrin Weidacker, Yingying Zhang, Naying He, Haiyan Jin, Weibo Chen, Valerie Voon, Richard A. E. Edden, Fuhua Yan

**Affiliations:** 1 Department of Radiology, Ruijin Hospital, Shanghai Jiaotong University School of Medicine, Shanghai, China; 2 Department of Functional Neurosurgery, Ruijin Hospital Shanghai Jiaotong University School of Medicine, Shanghai, China; 3 Department of Psychiatry, University of Cambridge, Cambridge, UK; 4 Philips Healthcare, Shanghai, China; 5 Department of Psychiatry, Ruijin Hospital Shanghai Jiaotong University School of Medicine, Shanghai, China; 6 Department of Radiology and Radiological Science, The Johns Hopkins University School of Medicine, Baltimore, MD USA

**Keywords:** Obsessive-compulsive disorder, Pathophysiology, H-MR spectroscopy, Glutamate-glutamine, GABA

## Abstract

**Background:**

Obsessive-compulsive disorder (OCD) is a relatively common and disabling psychiatric disorder whose pathophysiology is incompletely understood. In this study, we utilized magnetic resonance spectroscopy (MRS) in an effort to provide a better understanding of the role of brain gamma-aminobutyric acid (GABA) and glutamate in the pathophysiology of OCD. We hypothesized that beyond the separate effects of these neurotransmitter systems, a disruption in the balance between GABA and glutamate could be particularly relevant to OCD.

**Methods:**

We obtained MRS measures of GABA and glutamate concentrations in the anterior cingulate cortex from 23 adult patients with OCD and 20 sex- and age-matched healthy community volunteers. Established clinical rating scales were used to assess the severities of OCD, anxiety, and depression symptoms. Statistical analysis involved the assessment of patient-control group differences in the individual measures of GABA and glutamate, as well as in the ratio of the GABA to glutamate measures. Additionally, we explored whether differences in the MRS measures existed between two subgroups of patients formed according to the severity of their OCD symptoms. Finally, we assessed the relations of demographic and clinical variables to the MRS measures.

**Results:**

Patients with OCD displayed a higher estimated GABA level and a higher GABA to glutamate ratio than healthy participants, but no significant group differences were observed in the measure of glutamate. The MRS measures did not vary by subgroup and showed no correlations with demographic and clinical variables.

**Conclusions:**

These results indicate that GABA abnormalities within the anterior cingulate cortex contribute to the pathophysiology of OCD. The results fail to provide evidence that glutamate abnormalities alone are involved in adult OCD. Yet, it seems that a disruption in the balance between glutamate and GABA neurotransmission may have a particularly important role to play in OCD pathophysiology.

## Background

Obsessive-compulsive disorder (OCD) is a psychiatric disorder that afflicts approximately 1–3% of the general population. This disorder is characterized by recurrent intrusive thoughts (obsessions) and repetitive behaviors or mental acts (compulsions), the latter typically performed in response to obsessions or related anxiety [1]. OCD is a debilitating psychiatric disorder, ranked by the World Health Organization as one of the 10 most disabling illnesses in middle age due to loss of income and diminished quality of life [2]. Current treatment of OCD mainly consists of serotonergic medications, but a substantial portion of patients do no respond or respond insufficiently to these medications which makes it important to investigate the role of other neurotransmitters such as gamma-aminobutyric (GABA)and glutamate and their role in OCD. [2]. Despite its impact on patients, their families, and society as a whole, the pathophysiology of OCD remains incompletely understood. Clinical studies indicate that dysfunction of multiple neurotransmitter systems contributes to the pathophysiology of OCD. Initially, dysfunction of the central serotonergic system has been implicated on the basis of the observed therapeutic effects of selective serotonin reuptake inhibitors on OCD symptoms [2, 3]. Subsequent studies using positron emission tomography (PET) or single photon emission computed tomography (SPECT) have implicated additional neurotransmitters in the pathophysiology of OCD. For example, it has been reported that patients with OCD exhibit decreased striatal dopamine D2 receptor binding compared to healthy controls [4], which indicates that enhanced activity of the mesolimbic dopaminergic system could be involved in OCD pathophysiology. Also, patients with OCD have been characterized by increased glucose metabolism in the orbitofrontal cortex [5, 6]. Moreover, studies using magnetic resonance spectroscopy (MRS) have documented abnormalities in the levels of neurotransmitter-related metabolites in several distinct brain areas of patients with OCD [7], including decreased glutamate-glutamine (Glx) concentrations in the anterior cingulate cortex (ACC) [8] and increased gamma-aminobutyric (GABA) concentrations in the medial prefrontal cortex [9]. These clinical studies have contributed to the hypothesis that dysfunctions within cortico-striato-thalamo-cortical (CSTC) brain circuitry are fundamental to the pathophysiology of OCD.

These clinical studies have provided important clues about the pathophysiology of OCD, but the observed patient-control differences are usually small in size and do not exhibit high levels of consistency across studies [3, 10]. It seems that at least three factors make the interpretation of results difficult. First, most studies have examined only one neurotransmitter system, without taking into account the dynamic interactions occurring between different systems. It has been suggested, indeed, that the balance between the excitatory neurotransmitter glutamate and the inhibitor GABA is particularly relevant to the integrity of brain and cognitive function in both healthy and psychiatrically ill populations [11]. Second, OCD is usually considered as a simple homogeneous diagnostic entity, and not as a complex and etiologically and biologically hetergeneous disorder. Although no consensus exists about the number, nature, and identification of OCD subtypes, it would be informative to consider different subgroups of patients formed according to a basic clinical feature as illness severity or onset age. And third, in MRS studies it can be difficult to obtain accurate measures of the brain metabolites of interest due to a lack of sensitivity to the low concentrations present in the specific brain region examined and to spectral overlap with other signals and macromolecules. The present study was designed to overcome these three limitations.

In this study, we utilized MRS in an effort to provide a better understanding of the role of brain GABA and glutamate in the pathophysiology of OCD. To achieve this aim, we measured both GABA and Glx concentrations in the ACC of patients with OCD and matched healthy control participants. Moreover, we utilized the ratio of GABA to Glx in an effort to assess the balance between GABA and glutamate [12, 13]. As a key node in CSTC circuit, the ACC was chosen as the region of interest because this component of the CSTC seems especially relevant to the clinical symptoms of OCD, as well as to the disruption of cognitive and emotional functioning often seen in affected patients [14]. Furthermore, deep brain stimulation and ablative surgery of the ACC have been found to alleviate symptoms of severe, treatment-refractory cases of OCD [15], which exemplifies the important role the ACC plays in OCD.

Additionally, we examined whether differences in our measures of GABA and glutamate existed between two subgroups of patients formed according to the severity of their OCD symptoms. Finally, we employed a modified MRS technique to obtain reliable estimates of in vivo GABA and glutamate concentrations. Assuming that abnormalities of GABA and glutamate are indeed linked to the pathophysiology of OCD, we hypothesized that the patients with OCD and healthy controls would differ in the measured levels of GABA and glutamate in the ACC. If this hypothesis were to be confirmed, MRS-based measures of brain GABA and glutamate could potentially be developed into quantitative biomarkers to aid the diagnosis or treatment of patients with OCD.

According to our findings, the patients with OCD showed a higher GABA+ level and a higher GABA+/Glx ratio in the ACC compared to healthy control participants, but no significant group differences were observed in the level of Glx, which confirmed our hypothesis that the dynamic interplay between excitatory glutamate and inhibitory GABA neurotransmission is particularly involved.

## Materials and methods

### Participants

Study participants consisted of 23 patients with OCD and 20 healthy control (HC) participants between the ages of 24 and 50 years. Inclusion criteria for patients were: (1) diagnosis of OCD made by a licensed psychologist or psychiatrist on the basis of a clinical interview according to DSM-IV-TR (Diagnostic and Statistical Manual of Mental Disorders), (2) aged between 18 and 70 years, and (3) no visible MRI structural abnormality. Exclusion were: (1) diagnosis of intellectual disability or psychotic disorder, and (2) contraindications to MRI scanning. All participants were asked to stop medication for 24 h before scanning. Age- and sex-matched HCs who have no psychiatric or neurological disease were recruited from the local community through advertisements. Initially, the number of study participants recruited was higher than the number indicated above, but one patient and two control participants had to be excluded from analysis due to excessive motion artifacts during MRI scanning.

### Ethical statement

This study was approved by the ethics committee of our hospital. All participants provided written informed consent.

### Clinical assessments and subgrouping

Two experienced clinicians assessed the severity of OCD, anxiety, and depression of the patients using the Yale-Brown Obsessive-Compulsive Scale (Y-BOCS) [16, 17], Hamilton Anxiety Rating Scale (HAMA) [18], and the 17-item Hamilton Depression Rating Scale (HAMD-17) [19]. As indicated in the Introduction, the patient group was divided into two subgroups that differed from one another in OCD symptom severity, as measured by using the total Y-BOCS score: one subgroup of patients (*n* = 7) who displayed relatively ‘moderate’ OCD symptoms (Y-BOCS score < 25) and another subgroup (*n* = 16) who showed ‘severe’ symptoms (Y-BOCS score ≥ 25). Also, to explore potential effects of serotonergic medication treatment on our MRS measures, we compared one subgroup of patients (*n* = 5) who had never used serotonergic medication for their OCD to another subgroup of patients (*n* = 18) who had used medication (two types of selective serotonin reuptake inhibitors for at least 3 months) but had failed to show a clinical response.

### MRI scans

**Fig. 1:**
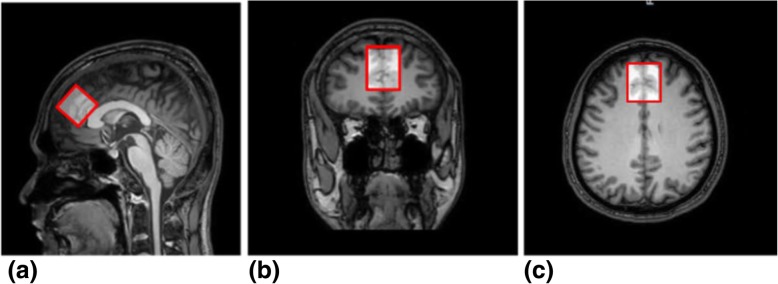
Position of MRS region of interest. A region of interest (ROI) with a size of 30 × 30 × 30 mm^3^ was created over the anterior cingulate cortex according to a high-resolution anatomic 3D T1WI MRI scan. The ROI is shown in (**a**) sagittal, (**b**) coronal, and (**c**) transversal orientations

All subjects were scanned using a 3.0 T MR scanner (Ingenia, Philips, Best, The Netherlands), with a fifteen-channel head coil. High-resolution 3D T1WI MRI images of the brain were obtained with the following parameters: repetition time/echo time (TR/TE) = 8.1/3.7 ms; isotropic voxel size = 1 mm^3^ by using the magnetization-prepared rapid gradient-echo sequence. The levels of GABA were measured using the MEGA-PRESS sequence [20], with the following parameters: TR/TE = 2000/68 ms, 14 ms editing pulses alternating at 1.9 ppm (“Edit-On”) and 7.46 ppm (“Edit-Off”) from the Larmor frequency of free water to separate the GABA molecule from other chemicals. The difference between the EDIT-ON and EDIT-OFF spectra yields the peaks affected by the editing pulses. The multiply optimized insensitive suppression train method was used to suppress the water signal and eight unsuppressed water averages were applied to quantify the concentration of GABA. The ACC was set as the volume of interest (VOI, 30 × 30 × 30 mm^3^, Fig. 1a-c), where the anterior grim is bounded in the genu of the corpus callosum, between the sulcus of the corpus callosum and the cingulate sulcus.

### Data processing

**Fig. 2:**
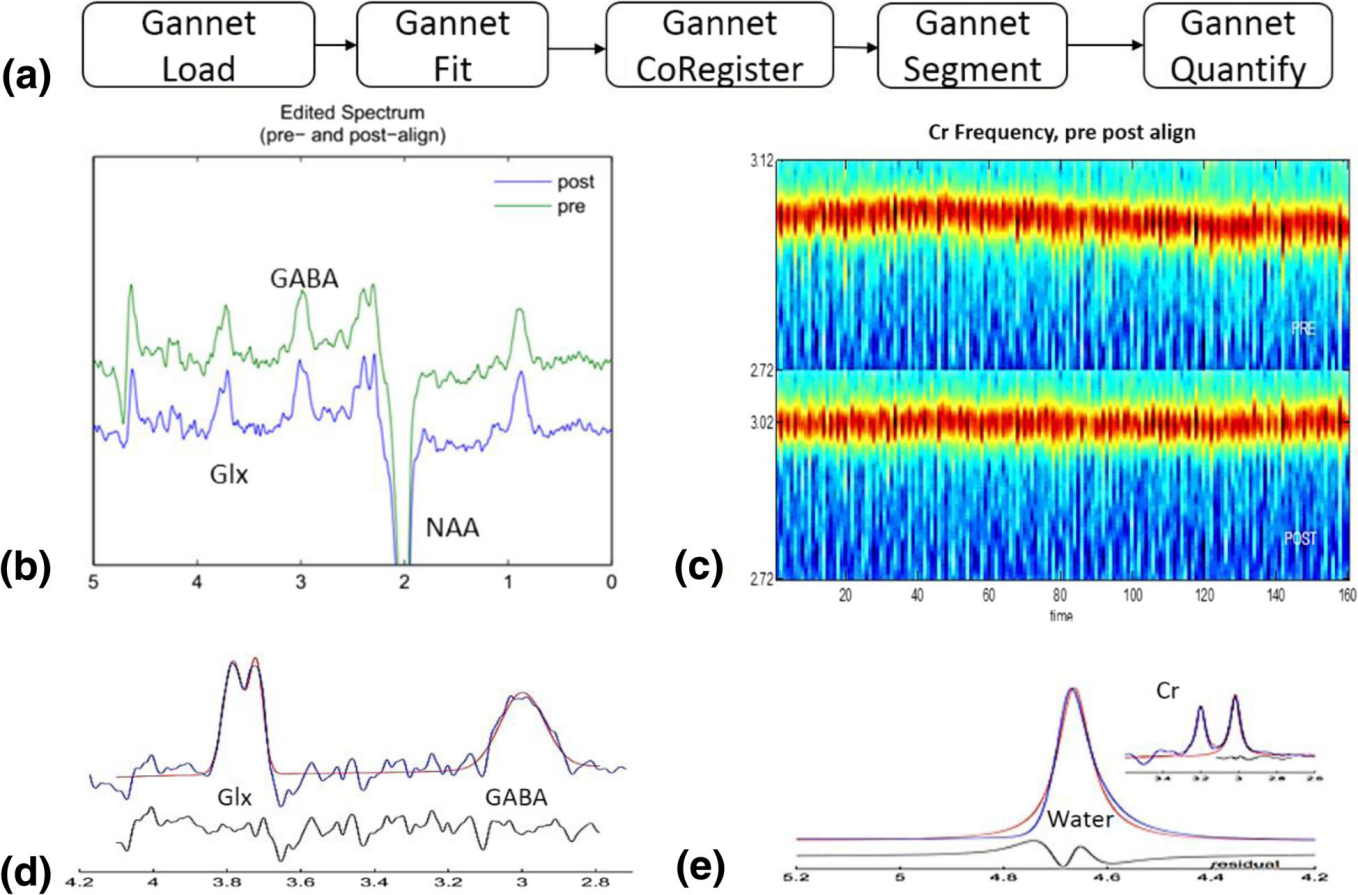
Flowchart of the MEGA-PRESS MRS data processing procedure and representative results of each step. (**a**). Schematic picture of the MEGA-PRESS MRS data processing procedure. (**b**) The processed GABA-edited difference spectrum (EDIT-OFF − EDIT-ON = DIFF) is the key output of the GannetLoad module. This plot shows the spectrum before frequency and phase correction (above in green) and the spectrum after frequency and phase correction (below in blue). (**c**) The Cr signal over the duration of the acquisition. The y-axis represents the frequency in ppm of the Cr signal. The spectra at each time point are presented as a vertical stripe in the image (color-coded according to signal intensity), so the Cr signal appears as a ‘hot’ stripe running through the image. In the lower half (POST), the result of frequency and phase correction is shown. (**d**) Model fitting of GABA+ and Glx spectrum peaks, representing the GABA+ signal modeling (GannetFit output). The blue line represents the actual edited spectrum while the overlaid red line is the model of best fit (using a simple Gaussian model by default). The residual is represented by the black curve below the modeling plot. (**e**) The modeling of the signal against which GABA is quantified, using the same color scheme as in (**d**). In our experiment, the unsuppressed water data were incorporated, so that the main spectrum is the water signal; the Cr signal is shown in an inset. The water signal was modeled as a mixed Gaussian-Lorentzian and served as the reference for GABA quantification

Due to fitting limitations, it’s unable to completely control for the potential contribution of macromolecules to the MRS signal detected. Therefore, in the remaining part of this paper, the signal detected at 3 ppm is labeled as GABA+ instead of GABA, indicating the potential contribution of these other compounds. The co-edited Glx peak was seen at 3.75 ppm and did not interfere with the GABA measurement. All GABA+ and Glx concentrations were quantified by using the MEGA-PRESS specialized tool Gannet v2.0 [21]. Levels of GABA+ and Glx were quantified by calculating the area under the curve; GABA+ levels were gauged via a GABA+/Water ratio. In Gannet, we used: (1) GannetLoad to process time-domain MRS data; (2) GannetFit to model the edited spectrum; (3) GannetCoRegister to generate a mask of the MRS voxel in T1-image space; (4) GannetSegment to derive gray matter (GM), white matter (WM), and cerebrospinal fluid (CSF) voxel fractions; and (5) GannetQuantify to calculate tissue-corrected GABA and Glx levels. A schematic picture of the data processing procedure is presented in Fig. 2.

The ratios of the integrals of the GABA+ and water signals, correcting for T1 and T2 relaxation times and partial volume effects, were used to calculate the water-scaled GABA+ concentration in mmol/L(mM) using the following formula [22]:

$$ \left[ GABA\right]=\frac{I_G}{I_W}\times \left[{H}_2O\right]\times {VIS}_W\times \frac{f_{GM}\times {R}_{W\_ GM}+{f}_{WM}\times {R}_{W\_ WM}+{f}_{CSF}\times {R}_{W\_ CSF}}{\left(1-{f}_{CSF}\right)\times {R}_G}\times MMcor $$

where *I*_*G*_ and *I*_*W*_ are the fitting integrals of GABA+ (G), and water (W) as determined by Gannet, [H2O] is the pure water concentration (55,550 mmol/L), *VIS*_*w*_ is a factor accounting for MR water visibility and tissue proton density (0.65), and *f*_*GM*_, *f*_*WM*_ and *f*_*CSF*_ are the fractions of water attributable to GM, WM and CSF respectively [22]. The relaxation attenuation factors are provided by the equation ${R}_{W\_y}=\mathit{\exp}\left[- TE/T{2}_{{}_W\_y}\right]\Big(1-\mathit{\exp}\left[- TR/T{1}_{W\_y}\right]$, where *T*1_*W* _ *y*_ and *T*2_*W* _ *y*_ are the T1 and T2 water compartment relaxation times. Similarly, *R*_*G*_ is the relaxation attenuation factor for GABA. The relaxation times used were as follows: GM water: T1 = 1331 ms, T2 = 110 ms; WM water: T1 = 832 ms, T2 = 79.6 ms; CSF: T1 = 3817 ms, T2 = 503 ms [23–25]; GABA: T1 = 1310 ms, T2 = 88 ms [26, 27]. MMcor is a macromolecular correction factor (0.45) given by the fraction of the GABA+ peak that is considered to reflect GABA [28].Gannet provides normalized residual fitting errors of GABA+, which can be interpreted quantitatively to assess measurement quality. Correspondingly, only spectra with a fitting error of GABA+ below 10% were included in the final analysis.

Each pixel in the 3D T1-weighted MRI images was segmented into GM, WM, and CSF using SPM8 [29]. VOIs were coregistered to the anatomical images using the “Re-creation of VOI” Matlab tool (as shown in Fig. 1). Tissue GM fractions were obtained by calculating the ratio of GM volume to the GM + WM volumes in the VOIs. The concentrations of GABA in the CSF were considered to be negligible [30].

### Statistical analysis

Independent-samples *t*-tests were conducted to assess mean differences between the OCD and HC groups in age, GABA+, Glx, GABA+/Glx ratio, and tissue composition (except for the GM data, which were analyzed using Mann-Whitney U tests). A chi-square test was performed to evaluate differences in the proportions of males and females between the OCD and HC groups. Independent-samples *t*-test were also used for all subgroup analyses within the patient group. Finally, Pearson correlation coefficients were computed to explore whether our MRS measures correlated with demographic and clinical variables. The statistical significance level was set at *p* < 0.05. SPSS v19.0 (IBM, Armonk, NY) was used to analyze the data.

## Results

### Sample characteristics

Table 1 presents demographic and clinical characteristics of the study participants. The OCD group did not differ significantly from the HC group in terms of sex composition and age, but not for education. The patients showed on average ‘severe’ OCD symptoms (mean Y-BOCS score = 29.0, *SD* = 7.6), ranging from mild (Y-BOCS score = 15) to extreme (Y-BOCS score = 40). The OCD symptoms of most patients became evident in late adolescence (mean age onset = 16.7, *SD* = 7.6). In addition to OCD, the patients displayed ‘moderate’ and ‘mild’ symptoms of anxiety (mean HAMA score = 18.4, *SD* = 11.1) and depression (mean HAMD-17 score = 14.1, *SD* = 8.5). Measures of tissue segmentation for both groups are provided in Table 2. There were no significant differences between the OCD and HC groups regarding tissue composition in the ACC ROI (all *p* > 0.078).

**Table 1:** Demographic and Clinical Characteristics of the OCD and HC Groups

	OCD (*n* = 23)	HC (*n* = 20)	*t or χ* ^2^	*df*	*p*
Mean age in years ± *SD*	31.7 ± 8.8	29.3 ± 5.6	1.061	37.846	0.295
Number of females/males	7/16	10/10	0.992	1	0.319
Mean age of OCD onset ± *SD*	17.2 ± 7.6	–			–
Mean years of education ± SD	16.8 ± 3.7	12.7 ± 3.2	−3.828	41	0.000*
Mean Y-BOCS ± *SD*	29.0 ± 7.8	–			–
Mean HAMA ± *SD*	18.4 ± 11.3	–			–
Mean HAMD-17 ± *SD*	14.1 ± 8.7	–			–

*HAMA* Hamilton Anxiety Rating Scale, *HAMD-17* 17-item Hamilton Depression Rating Scale *HC* healthy control, *OCD* obsessive-compulsive disorder, Yale-Brown Obsessive-Compulsive Scale

*p < 0.05

**Table 2:** ACC Metabolite Levels and Tissue Composition in the OCD and HC groups

	OCD (*n* = 23)	HC (*n* = 20)		
**MRS metabolite**	**Mean** ***± SD (iu***^***+***^***)***	**Mean** ***± SD (iu)***	***t(df)***	***p***
GABA+	2.41 ± 0.59	2.10 ± 0.35	2.05 (41)	0.047*
Glx	2.10 ± 0.49	2.23 ± 0.38	−0.97 (41)	0.34
GABA+/Glx Ratio	1.18 ± 0.30	0.96 ± 0.22	2.70 (41)	0.01*
**Tissue composition**	**Mean Percentage** ***± SD***	**Mean** ***± SD***	***t(df)***	***p***
Cerebral spinal fluid	0.17 ± 0.02	0.16 ± 0.02	1.82 (41)	0.0781
Gray matter	0.52 ± 0.03	0.52 ± 0.03	/	0.752
White matter	0.31 ± 0.04	0.32 ± 0.03	1.59 (41)	0.3431

^+^iu, institutional units; *ACC* anterior cingulate cortex, *GABA* gamma-aminobutyric acid, *Glx* glutamate-glutamine, *MRS* magnetic resonance spectroscopy, *HC* healthy control, *OCD* obsessive-compulsive disorder

*significant between-group difference (*p < 0.05*)

### Differences between OCD and HC groups

**Fig. 3:**
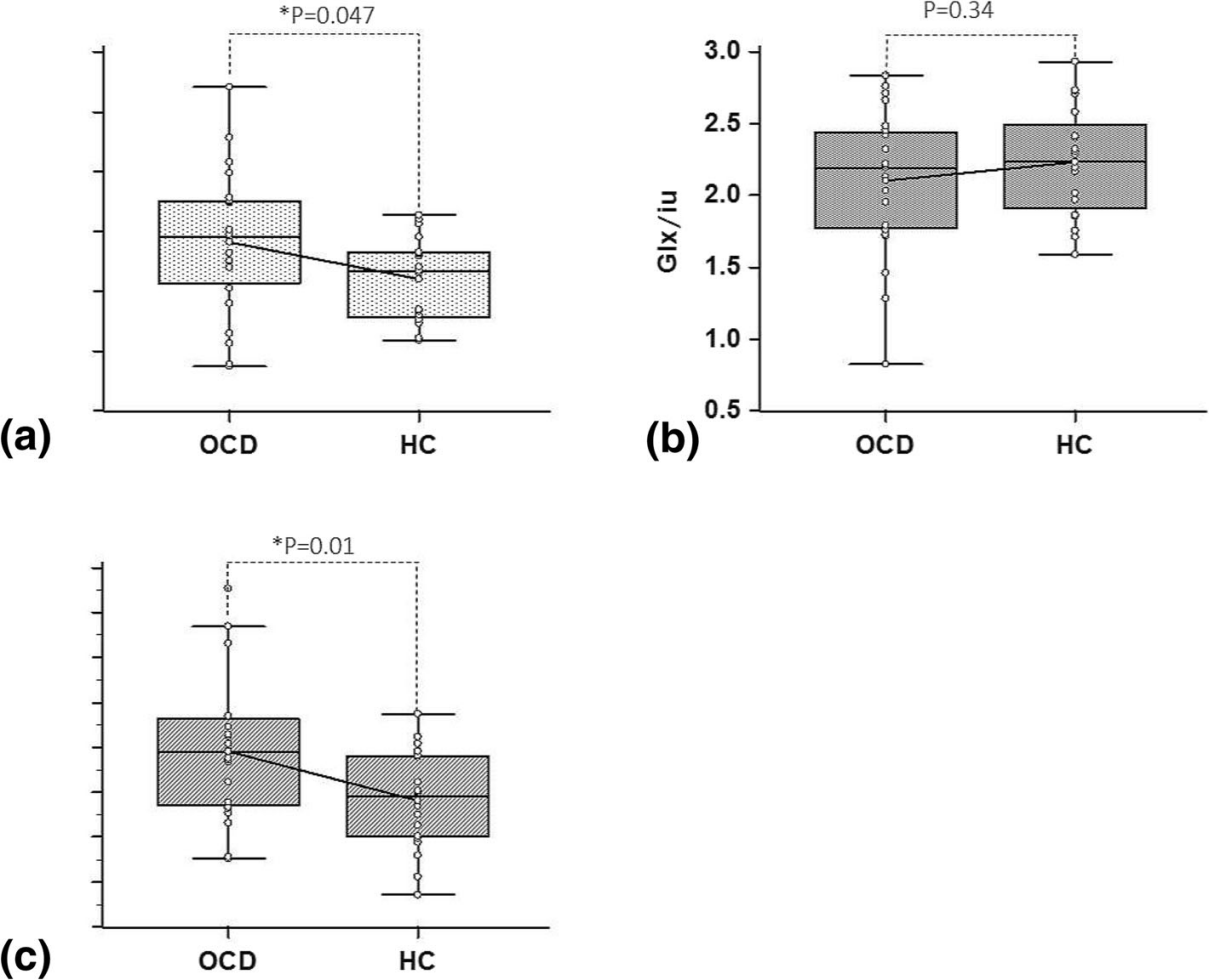
Comparison between OCD and HC groups in **a** GABA+, **b** Glx and **c** GABA+/ Glx values. Significant differences between the OCD and HC groups were observed in the level of GABA+ and the GABA+/Glx ratio. The OCD group showed a higher level of GABA+ and a higher GABA+/Glx ratio than the control group. By contrast, no significant differences between the OCD and HC groups were observed in the level of Glx

Significant differences between the OCD and HC groups were observed in the level of GABA+ and the GABA+/Glx ratio (As shown in Fig. 3 and Table 2). The OCD group showed a higher level of GABA+ and a higher GABA+/Glx ratio than the control group (Table 2), with the size of the between-group differences being medium (Cohen’s *d* = 0.64) and large (Cohen’s *d* = 0.84), respectively. By contrast, no significant differences between the OCD and HC groups were observed in the level of Glx.

### Differences between OCD subgroups

Patients classified as having either relatively ‘moderate’ OCD or ‘severe’ OCD did not differ significantly from one another on the MRS measures (Table 3). Similarly, patients who had never received serotonergic medication treatment did not differ significantly in their levels of GABA+ and Glx compared to patients who had a history of medication treatment (Table 3). However, never medicated patients exhibited a significantly larger GABA+/Glx ratio than patients with a history of serotonergic medication treatment.

**Table 3:** MRS measures as a function of patient subgroups

MRS metabolite	un-medicated OCD (*n* = 5)	refractory OCD (*n* = 18)	*t(df)*	*p*	severe OCD (*n* = 16)	moderate OCD (*n* = 7)	*t(df)*	*p*
GABA+	2.43 ± 0.23	2.40 ± 0.66	0.093 (21)	0.927	2.37 *± 0.61*	2.48 *± 0.58*	0.404 (21)	0.69
Glx	1.93 ± 0.42	2.15 ± 0.51	0.903 (21)	0.377	2.12 ± 0.56	2.06 ± 0.32	0.258 (21)	0.80
GABA+/Glx Ratio	1.31 ± 0.34	1.15 ± 0.29	1.116 (21)	0.277	1.16 ± 0.30	1.22 ± 0.32	0.436 (21)	0.667

*ACC* anterior cingulate cortex, *GABA* gamma-aminobutyric acid, *Glx* glutamate-glutamine, *HC* healthy control, *MRS* magnetic resonance spectroscopy, *OCD* obsessive-compulsive disorder

### Correlations of MRS measures with demographic and clinical variables

The MRS measures showed no significant correlations with the demographic and clinical variables under study (Table 4).

**Table 4:** Correlations of MRS measures with demographic and clinical variables within the patient group (*N* = 23)

*MRS metabolites*	Age	Age of Onset	Y-BOCS	HAMA	HAMD-17
GABA+ *r*(*p*)	−0.264 (0.224)	0.307 (0.154)	−0.307 (0.154)	−0.033 (0.881)	0.001 (0.995)
Glx *r*(*p*)	−0.144 (0.512)	0.309 (0.151)	0.007 (0.976)	0.01 (0.964)	0.071 (0.746)
GABA+/Glx Ratio *r*(*p*)	−0.122 (0.578)	−0.055 (0.805)	− 0.267 (0.218)	−0.071 (0.749)	− 0.145 (0.509)

*GABA* gamma-aminobutyric acid, *Glx* glutamate-glutamine, *HAMA* Hamilton Anxiety Rating Scale,

*HAMD-17* 17-item Hamilton Depression Rating Scale, *OCD* obsessive-compulsive disorder

## Discussion

In this study, patients with OCD showed a higher GABA+ level and a higher GABA+/Glx ratio in the ACC than healthy control participants, but no significant group differences were observed in the level of Glx. The former result supports the hypothesis that GABA abnormalities in the ACC are involved in the pathophysiology of OCD. Furthermore, the higher GABA+/Glx ratio observed in patients seems to confirm our hypothesis that the dynamic interplay between excitatory glutamate and inhibitory GABA neurotransmission is particularly involved. Indeed, according to the obtained effect size estimates, the diagnostic sensitivity of this ratio measure was better than the measure of GABA alone. Finally, the present results fail to support the hypothesis that ACC glutamate abnormalities alone, as indexed by Glx levels, play a significant role in OCD pathophysiology.

Our results are difficult to compare directly to previous studies on GABA levels in patients with OCD because of methodological and patient sample differences. Furthermore, previous MRS studies focused on the prefrontal cortex (PFC), and not on the ACC. For example, Simpson et al. [31] found decreased GABA/W levels in the medial PFC of adult patients with OCD when compared to matched healthy participants. By comparison, these researchers did not detect group differences in GABA/W levels in the dorsolateral PFC. Other studies have reported that plasma levels of GABA are decreased in patients with OCD [32]. Despite marked study differences, the present results also indicate that GABA abnormalities within the CSTC circuit are linked to OCD pathophysiology. An important goal for further research is to determine whether our finding of increased GABA levels in the ACC of patients with OCD bears a relationship to decreased GABA levels in the medial PFC reported in other studies.

The finding that the patients with OCD did not differ from healthy participants in the level of Glx in the ACC is also in agreement with previous studies [26, 29] and, specifically, with the findings of Brennan et al. [33], who reported the same finding in a patient sample similar to our sample with respect to age, sex, and severity of OCD symptoms. These researchers also question whether glutamate abnormalities in the ACC are fundamental to OCD pathophysiology, but leave room that these abnormalities could play a role in the early course of the disorder [30] or in certain subtypes of OCD. Alternatively, our study results indicate that glutamate abnormalities alone may not be directly involved, but that the interaction of this excitatory neurotransmitter with GABA could well have a significant pathophysiological role to play in OCD.

The results of this study, however, should be qualified because the observed GABA+ levels and GABA+/Glx ratios neither differentiated patients with severe OCD from patients with relatively moderate OCD nor correlated with our continuous variables of clinical symptom severity. Moreover, given the nonexperimental nature of case-control studies, the possibility cannot be completely excluded that the differences in the MRS measures between the patients with OCD and healthy participants stem from factors other than the disease state of interest. Although both groups were similar to each other with respect to sex and age, and serotonergic medication treatment was associated with decreased, and not with increased, GABA+/Glx ratios, the patients with OCD could have differed from the community volunteers on numerous other variables that potentially have affected the MRS measures but that were not assessed and controlled for in the study. For example, both groups could have differed from one another in cognitive function or socioeconomic background. Moreover, the patients could have differed from the community volunteers on variables that are not directly linked to the pathophysiological process of interest but that are likely to accompany the clinical diagnosis of OCD, such as treatment and hospitalization along with the psychological and psychosocial consequences of having received the formal diagnosis of the psychiatric disorder. In addition, SSRIs may take 1 week till they have a noticeable effect and the effect should also not immediately decay if the patients do not use them for 24 h. We cannot entirely exclude the SSRIs effect in the medicated patients, which is a limitation [34, 35]. The healthy controls were matched for age, gender, but not for education and the small sample size is also a limitation that should be considered in our future studies. Our study results and conclusions, therefore, should be regarded as tentative and preliminary until they have been substantiated by further research.

## Conclusions

This MRS study indicates that GABA abnormalities within the ACC contribute to the pathophysiology of OCD. Also, in line with previous MRS studies, the results fail to provide evidence that glutamate abnormalities alone are involved in adult OCD. New tentative evidence is provided, however, that a disruption in the balance between GABA and glutamate neurotransmission has a particularly important role to play in OCD pathophysiology. However, because of study limitations, additional research is required to validate the present observations and interpretations.

## Data Availability

The data that support the findings of this study are available from the corresponding author upon reasonable request.

## Abbreviations

^1^H-MRS: proton MRS
ACC: Anterior cingulate cortex
CSF: Cerebrospinal fluid
CSTC: Cortico-striato-thalamo-cortical
DLPFC: Dorsolateral prefrontal cortex
GABA: Gamma-aminobutyric acid
Glx: Glutamate-glutamine
GM: Gray matter
HAMA: Hamilton Anxiety Rating Scale
HAMD-17: 17-item Hamilton Depression Rating Scale
HC: Healthy control
MEGA-PRESS: MEGA-Point Resolved Spectroscopy technique
mPFC: medial prefrontal cortex
MRS: Magnetic resonance spectroscopy
OCD: Obsessive-compulsive disorder
ROI: Region of interest
TE: echo time
TR: repetition time
WM: White matter
Y-BOCS: Yale-Brown Obsessive-Compulsive Scale
